# Response: Commentary: Emotion Perception in Members of Norwegian Mensa

**DOI:** 10.3389/fpsyg.2019.02337

**Published:** 2019-10-17

**Authors:** Jens Egeland

**Affiliations:** ^1^Division of Mental Health and Addiction, Sykehuset i Vestfold, Vestfold Hospital Trust, Tønsberg, Norway; ^2^Department of Psychology, University of Oslo, Oslo, Norway

**Keywords:** EmoBio test, emotion recognition, Mensa, social cognition, control group

My colleague Vaskinn (2019) published recently a commentary on my study on Emotion Perception in Norwegian Mensa (Egeland, 2019). My study is the first to actually test social cognition in Mensa Members, although self-ratings have been published previously (Karpinsky et al., 2018). Vaskinn questions the validity of the finding that Mensa members score superior to community controls on the EmoBio test of emotion recognition. When comparing the Mensa-study results to her own healthy controls in studies of patients with schizophrenia (Vaskinn et al., 2016) and bipolar disorder (Vaskinn et al., 2017) she points to a difference between the scores of her and my control groups. If the Mensa study had used her control group, the Mensa sample would not have performed better, in fact they performed numerically lower than her control group.

Her point is important, and that is why all research has to be replicated with new community samples as well as new experimental groups. There are, however, two possible reasons for the difference in scores. The first is related to the distribution of age in her study compared to the Mensa study. The mean age was comparable (around 30 years) but the standard deviation in the Mensa study was double the size of her controls (15.6 years compared to 8.1). The median age was 36. The distribution in the Mensa sample was normally distributed (skewness 0.615, kurtosis 0.397).

Most studies show that emotion recognition is reduced with increased age, in fact Kessels and colleagues found a decay beyond the age of 35 (Kessels et al., 2014). Analyzing only persons below the age of 46 (i.e., more than two standard deviations above the mean age of Vaskinn's sample), increased the EmoBio scores of the remaining control sample from 0.816 to 0.833 (s.d. 0.10). The control sample was then reduced from 101 to 79 participants. The Mensa sample, reduced from 63 to 53 participants, had an unchanged score of 0.857 (s.d. 0.08). A perfect score on all 22 items in the test would have given a score of 1.

Although this reduce the difference between Vaskinn's controls and the controls of the Mensa study, the Vaskinn control's mean of 0.87 is still above the controls of the Mensa study. The Vaskinn controls also performed above the control subjects of Couture et al. (2010), who introduced the scoring method that both she and I have used. In the Couture et al. study the healthy controls scored 0.84 for this young sample (mean age 27) which is similar to my controls younger than 46 years.

This leads us to discussing an important question for much research in psychiatry, i.e., the recruitment of controls. In the Vaskinn studies the controls were recruited through national statistical records, invited by letter to participate and screened with an interview to capture symptoms of severe mental illness.They were excluded from the study if mental, neurological, or somatic disorder was confirmed or suspected. It is unclear how many were invited and declined participation, or how many were refused participation due to the exclusion criteria. The subjects were tested individually.

The community sample in the Mensa study was graduate students from a local high school and employees in a national governmental agency. There were no exclusion criteria. They were tested in a group session, everyone was given a scoring sheet and a pen. The testing was incorporated in a guest lecture on psychology in the High School and on social abilities in the governmental agency. Everyone present at the lectures went through the testing, but were free to hand in the scoring sheet afterwards. We were not aware of any person declining this. The same procedure was used in the Mensa group. As the test consists of 22 film clips incorporated in a Power-Point display, it is ideal for group presentations. That there were no direct personal communication between the test-leader and the participant could have had an effect on motivation, but there is no reason to expect an interaction in the sense that it would affect only one group and not the other. But, of course, this has to be checked in future research. There is no a priori reason to consider the two community samples at risk for a specific impairment in social cognition. In fact, comparing the Mensa participants to a sample of employees in this governmental agency could have been criticized as unjust for the Mensa sample. Although they varied from secretaries to department directors, they nevertheless had competed for their professional position. There could be persons with mental disorders or social disabilities present, but this must be considered part of ordinary life challenges in the sense that no one were on sick leave.

The controls in the Mensa study seem to behave as expected from other studies of emotion recognition applying other tests than EmoBio, indirectly testifying to the validity of the findings: There was the expected age and gender effect, which was not found in Vaskinn's sample.

The most probable cause of the discrepancy between the two control samples must be due to recruitment. It is always a risk that control samples may be super-groups when exclusion criteria is strict. An even larger risk is that of skewed recruitment, i.e., that persons questioning their social abilities will decline voluntary participation when invited by letter. In the Mensa-study there were minimal risk of this since all of the subjects present at the lectures, actually participated in the study.

Vaskinn et al.'s original study (2016) showed a very robust impairment in emotion recognition among patients with schizophrenia. These findings would be significant even if a new control sample would score similar to the control sample of the Mensa study. The effect size in the next study (Vaskinn et al., 2017), applying the original control sample in a comparison to patients with bipolar disorder, would neither be significant if compared to the Mensa study controls nor to the Couture et al. (2010) controls. This emphasize the need for replications with new controls.

## Author Contributions

The author confirms being the sole contributor of this work and has approved it for publication.

### Conflict of Interest

The author declares that the research was conducted in the absence of any commercial or financial relationships that could be construed as a potential conflict of interest.
